# tRNA-derived fragments as New Hallmarks of Aging and Age-related Diseases

**DOI:** 10.14336/AD.2021.0115

**Published:** 2021-08-01

**Authors:** Ya Yuan, Jiamei Li, Zhi He, Xiaolan Fan, Xueping Mao, Mingyao Yang, Deying Yang

**Affiliations:** ^1^Institute of Animal Genetics and Breeding, Sichuan Agricultural University, Chengdu 611130, Sichuan, China.; ^2^Farm Animal Genetic Resources Exploration and Innovation Key Laboratory of Sichuan Province, Sichuan Agricultural University, Chengdu 611130, Sichuan, China.

**Keywords:** tRFs, regulation mechanisms, aging, age-related diseases

## Abstract

tRNA-derived fragments (tRFs), which are non-coding RNAs produced via tRNA cleavage with lengths of 14 to 50 nucleotides, originate from precursor tRNAs or mature tRNAs and exist in a wide range of organisms. tRFs are produced not by random fracture of tRNAs but by specific mechanisms. Considerable evidence shows that tRFs are detectable in model organisms of different ages and are associated with age-related diseases in humans, such as cancer and neurodegenerative diseases. In this literature review, the origin and classification of tRFs and the regulatory mechanisms of tRFs in aging and age-related diseases are summarized. We also describe the available tRF databases and research techniques and lay a foundation for the exploration of tRFs as biomarkers for the diagnosis and treatment of aging and age-related diseases.

## 1. Introduction

Aging is a process in which various functions in organisms are generally weakened and the abilities of organisms to resist environmental damage and restore physiological balance are gradually reduced. Studies involving various model organisms (from yeast to mammals) have revealed that the mechanism of lifespan regulation has been highly conserved throughout evolution [1]. Aging is not a disease but is a major cause of age-related diseases, such as cancers, cardiovascular diseases and neurological diseases [1, 2]. Aging and age-related diseases are complex processes affected by many factors, such as genes and the environment. Recent research has found that aging promotes increases in methyl malonic acid (MMA) levels in the blood, enabling cancer cells to migrate, invade, survive and progress and shortening survival in a cancer-related manner [3, 4]. Non-coding RNAs (ncRNAs), including long ncRNAs (lncRNAs), circular RNAs (circRNAs), microRNAs (miRNAs) and tRNA-derived fragments (tRFs), also play important regulatory roles in the processes of aging and age-related diseases. tRFs are cleaved from tRNAs under specific conditions (such as hypoxia stress) by endonucleases and angiopoietin (angiogenin, ANG). According to their cleavage sites and lengths, fragments derived from tRNAs are divided mainly into two types: tRNA halves (tiRNAs, tiRs) and tRFs [5, 6]; however, tiRs can also be classified generally as tRFs.

tRFs exist in archaea, bacteria, eukaryotes and the plant *Arabidopsis thaliana* [7-10]. With the development of high-throughput sequencing technology, an increasing number of tRFs have been identified. Studies have shown that tRFs can participate in the regulation of gene expression through various mechanisms. For example, tRF-3006 can bind nucleotides 56-67 of host cell tRNA-Lys, which constitute the primary binding site of HIV genomic RNA and play a role as a primer in the process of reverse transcription [11]. tRF-3006 can also bind Argonaute 2 (Ago2) and thereby silence reporter genes [11]. In addition, Shao *et al* [12] found that a class of RNA fragments derived from tRF-Leu-CAG is highly expressed in non-small-cell lung cancer (NSCLC), is related to tumour stage, and can enhance lung cancer cell proliferation by regulating a serine/threonine kinase (AURKA). tRFs have also been used as cancer biomarkers in some studies; for example, tDR-000620 has been used as a target for disease prediction and recurrence in triple-negative breast cancer patients [13]. Furthermore, tRFs are differentially expressed (DE) in *Drosophila melanogaster* of different ages (3 days old and 30 days old) [14]. Sequencing of *Plasmodium falciparum* has revealed that there are three main types of tRFs, namely, 5'ptRFs, 3'ptRFs and mid-ptRFs, in this species, of which 5'ptRFs are the most abundant (mid-ptRFs are cut from the anticodon ring, including the 5' end of tRNA, which is equivalent to the 5'tiR in the traditional classification of tRFs) [15]. These results suggest that tRFs may regulate aging and age-related diseases in organisms through various mechanisms.

Moreover, tRFs such as Gly-tRFs and tRF78576 have been widely found to play roles in non-age-related diseases. For example, Gly-tRFs can promote alcoholic liver injury and steatosis, but interference with the C3 activation step and treatment with Gly-tRF inhibitors can be used to alleviate alcoholic fatty liver disease (AFLD) [16]. tRF78576 exerts a regulatory effect on adipocytes by directly targeting the Kruppel-like factor (KLF) family, which is involved in cell proliferation, apoptosis, differentiation and embryonic development [17]. Thus, tRFs are involved in the regulation of a wide range of diseases and have potential application value.

This paper reviews the origin and classification of tRFs and the regulatory mechanisms of tRFs in aging and age-related diseases. It also describes the available tRF databases and related research techniques. The article thus lays a foundation for the exploration of tRFs as biomarkers for the diagnosis and treatment of aging and age-related diseases.

**Figure 1.**
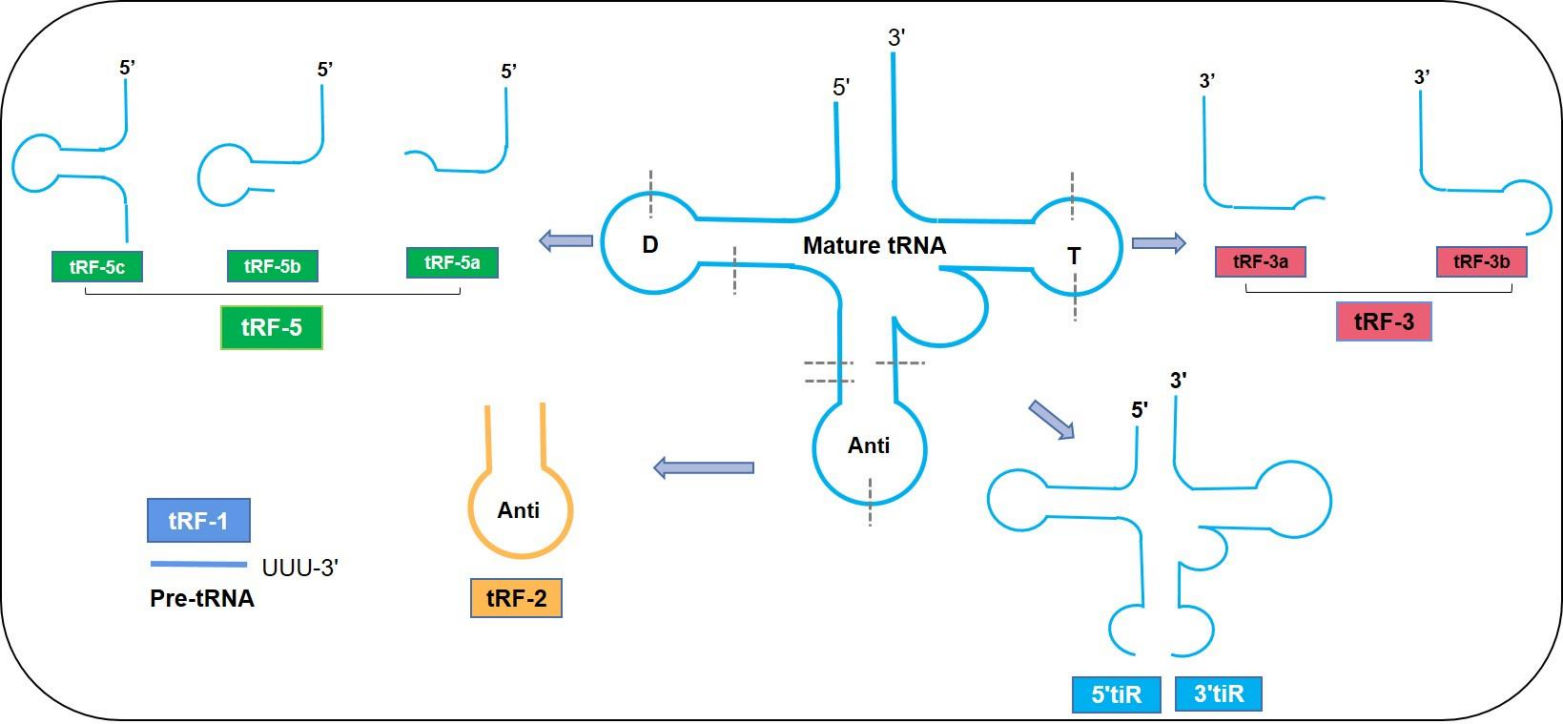
Classification of tRFs.

## 2. Biogenesis and regulatory mechanisms of tRFs

### 2.1. Origins and classification of tRFs

In recent years, tRFs have become a popular new research topic in the field of small ncRNAs (sncRNAs). tRFs are derived from tRNAs with multiple endonuclease cleavage sites, formed by specific mechanisms and widely expressed in cells and organisms [5]. tRFs can be divided into tRF-1s, tRF-2s, tRF-3s and tRF-5s (Fig. 1). Studies have shown that tRF-1s, tRF-3s, and tRF-5s are produced via cleavage by endonucleases, such as Dicer, while tiRs can be produced via cleavage by the *E. coli* nucleases Prrc, colicin D and ribonuclease E5; the yeast nuclease Rny1p; and the mammalian protein ANG [7, 18]. tRF-1s are derived from the 3' ends of tRNA precursors [19, 20], and the ends are all U bases. tRF-2s are newly discovered tRFs that include tRNAGlu, tRNAAsp, tRNAGly and tRNATyr; tRF-2s are decomposed by the anticodon subrings of tRNAs, excluding the structures of the 5' and 3' ends [21]. tRF-3s (18-22 nt) are derived from the 3' terminal T-ring structures of mature tRNAs [11, 22-24] such that the tail end of each tRF-3 contains the specific CCA structure of the mature tRNA 3' end. tRF-5s (14-30 nt) originate from the 5' terminal D-ring structures of mature tRNAs and are found mainly in mammalian cells, plant cells and yeast fission cells [25-28]. According to the specific cutting position, tRF-3s are subdivided into the following two types: tRF-3as (which are cut near the 18th base from the 3' end) and tRF-3bs (which are cut near the 22nd base from the 3' end). tRF-5s are cut before the anticodon ring and can be subdivided into the following three types [9]: tRF-5as (which are cut near the 14th-16th base from the 5' end), tRF-5bs (which are cut near the 22nd-24th base from the 5' end) and tRF-5cs (which are cut near the 28th-30th base from the 5' end) (Fig. 1). tRF-5s are found mostly in the nucleus, while tRF-3s and tRF-1s are found mostly in the cytoplasm [9, 23]. Under pressure stress, mature tRNAs split at the anticodon ring, producing tRFs to form tRNA semi-molecules, also known as tiRs (31-40 nt); these tiRs are divided mainly into 3'tiRs and 5'tiRs [29].

### 2.2. Regulatory mechanisms of tRFs

**Figure 2.**
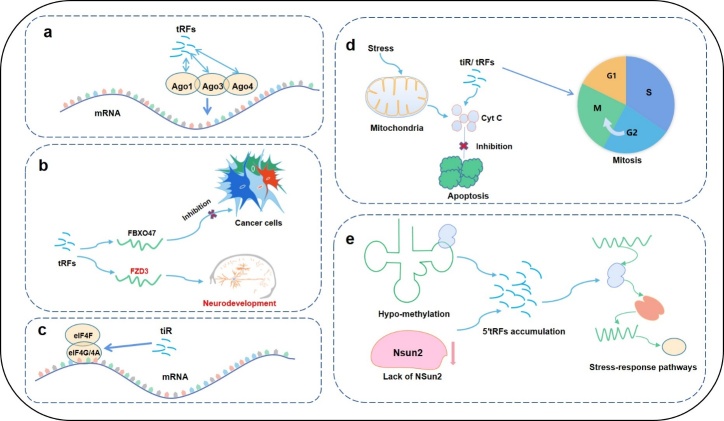
Regulatory mechanisms of tRFs. A) tRFs interact with proteins to regulate gene expression. B) tRFs target genes to inhibit the proliferation of cancer cells and affect early neural development. C) tRFs affect protein synthesis by regulating protein levels and inhibiting translation initiation and progression. D) tRFs participate in cell cycle regulation by promoting the progression of prostate cancer cells from the G2 phase to the M phase and inhibiting the formation and activity of apoptotic bodies. E) tRFs are associated with the post-transcriptional methylation of tRNAs. Hypo-methylation of tRNA or lack of NSun2 enables the accumulation of 5′tRFs, and these 5′tRFs activate stress-response pathways.

Studies investigating tRFs have identified many mechanisms of tRFs [30-33]. The main functional mechanisms of tRFs and tiRs are as follows:

(1) tRFs interact with proteins to regulate gene expression; thus, tRFs participate in important physiological processes, such as cell proliferation and DNA damage [34, 35]. A recent study reported that tRF-CU1276 has the functional characteristics of a miRNA in B-cell lymphoma; for example, tRF-CU1276 binds the AGO protein and inhibits mRNA transcription through a specific sequence [34] (Fig. 2A). tRFs compete with carcinogenic transcripts such as EIF4G1, ITGB4, and AKT1 to bind the RNA-binding protein YBX1 in order to regulate the stability of tumour gene transcripts and inhibit the proliferation and metastasis of cancer cells [36]. Other studies have shown that some tRF-3s in cells may target HIV-1 through the RNA interference (RNAi) pathway, which is also related to the Ago2 protein [11]. tRFs also play roles in viruses [37-39], inhibit apolipoprotein E receptor-2 (APOER2) expression, and promote RSV replication [39].
(2) tRFs can target genes and regulate gene expression. tRFs can regulate cancer cells by directly regulating tumour suppressor genes. For example, TRF-3019a inhibits gastric cancer cell proliferation by directly regulating the tumour suppressor gene *FBXO47* [40] (Fig. 2B). Furthermore, tRFs can target important factors in signalling pathways and affect the regulation of these pathways. For instance, 5'-tiRNAVal can target human Frizzled homologue 3 (*FZD3*) to inhibit the Wnt/β-catenin pathway and inhibit the proliferation and development of tumour cells in breast cancer [41-43]. In addition, the binding of tRFs and *FZD3* affects early neural development [41-43] (Fig. 2B).
(3) tRFs affect protein synthesis by regulating protein levels and inhibiting translation initiation and progression. For example, tiRs can inhibit translation initiation by binding the eiF4G/A complex [44] (Fig. 2C). VAL-tRF can inhibit protein translation by reducing the conserved residues in tRNAs via interference with peptidyl transferase activity through a process that does not require complementary targets in mRNAs [45-47]. The tRNA-derived small RNA LeuCAG3'tsRNA, which is produced from the Leu-CAG tRNA 3' terminal region, can regulate human ribosomal production and protein synthesis by maintaining the levels of ribosomal protein S28 (RPS28) [48].
(4) tRFs participate in cell cycle regulation. The expression level of tRF-1001 is positively correlated with cell proliferation in prostate cancer cell lines, and overexpression of tRF-1001 can promote the progression of prostate cancer cells from the G2 phase to the M phase [5]. tRFs can also interact with cytochrome C (Cyt C) under hypertonic stress [49, 50]. ANG-mediated tiRs can interact with Cyt C released by mitochondria to form a ribonucleoprotein complex and thereby inhibit the formation and activity of apoptotic bodies [49, 50] (Fig. 2D).
(5) tRFs are associated with the post-transcriptional methylation of tRNAs [51]. Hypo-methylation of tRNA or a lack of NSun2 (a cytosine-5 RNA methyltransferase) allows the accumulation of 5′tRFs. These 5′tRFs activate stress-response pathways, leading to reductions in protein translation rates, decreases in cell size and increases in cell death [51]. Abnormal growth and neurodevelopmental deficiencies have been observed in mice and humans [51] (Fig. 2E).

Further studies have also revealed additional regulatory mechanisms. For example, some studies have shown that tRFs are enriched in sperm cells, are transported to zygotes and affect gene expression through elements in the zygote genome; however, the exact mechanism is unclear [52, 53]. Kyoto Encyclopedia of Genes and Genomes (KEGG) and Gene Ontology (GO) analyses can be performed to analyse the regulation of tRF gene expression. In the future, the development of new technologies may aid in exploitation of the functions and mechanisms of tRFs and enable identification of additional tRF mechanisms. Such progress will further promote research investigating the roles of tRFs in the aging process.

## 3. tRFs in human and animal aging

**Figure 3.**
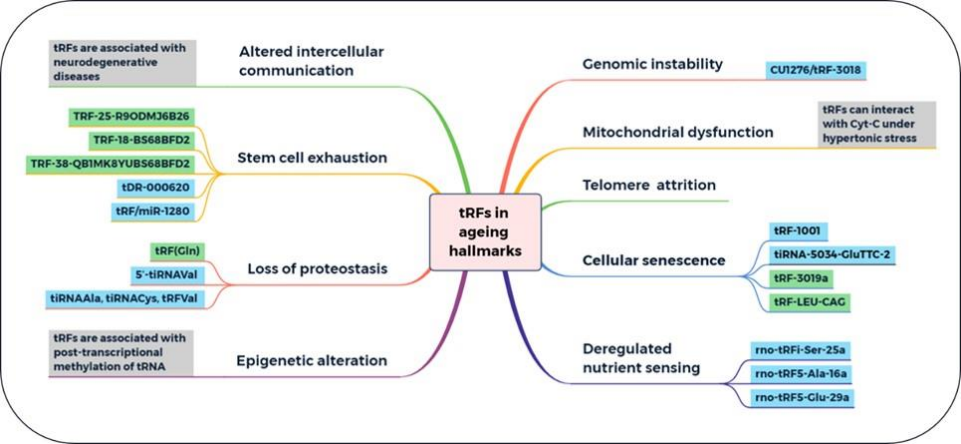
Hallmarks of aging and tRFs associated with aging hallmarks. Blue box, upregulated in disease; green box, downregulated in disease. Aging features the following 9 hallmarks: loss of proteostasis, stem cell exhaustion, altered intercellular communication, deregulated nutrient sensing, cellular senescence, telomere attrition, mitochondrial dysfunction, genomic instability and epigenetic alterations. Researchers have found that tRFs are associated with many of these hallmarks.

Aging is a complex process that features 9 hallmarks (loss of proteostasis, stem cell exhaustion, altered intercellular communication, deregulated nutrient sensing, cellular senescence, telomere attrition, mitochondrial dysfunction, genomic instability, and epigenetic alteration) [1]. These hallmarks must meet three criteria. First, these hallmarks should manifest during normal aging; second, experimental amelioration of the hallmarks should retard the normal aging process and, hence, extend healthy lifespan; and third, experimental aggravation of the hallmarks should accelerate aging [1]. Research investigating tRFs has typically focused on aging-related diseases but not on a specific mode of action in normal aging. Combined with the aging hallmarks, tRFs play some roles in aging (Fig. 3, Table 1).

Numerous tRFs have been found to be DE in humans and in various model organisms, especially model organisms of different ages; however, tRF expression in diseased tissues compared with adjacent normal tissues in the same organism has not been studied, nor have the deeper mechanisms. Model organisms are important tools in research on the roles and regulatory mechanisms of tRFs. Studies investigating tRFs in humans and model organisms have shown that tRFs have differential expression patterns in the contexts of human age-related disease and physiological senescence.

**Table 1:** tRFs associated with aging hallmarks.

Aging hallmarks	tRF name	Type	Targets	Mechanism of action	Dysregulation	Ref.
Cellular senescence	tiRNA-5034-GluTTC-2	tiR-5	-	Downregulated in cancer tissue, and the expression level is inversely proportional to the tumour size	Down	[54]
	tRF-3019a	tRF-3	*FBXO47*	Regulates the tumour suppressor gene *FBXO47*	Up	[40]
	tRF-1001	tRF-1	-	Regulates cell proliferation	Down	[5]
	tRF-LEU-CAG	tiR-5	AURKA	Related to the proliferation of cancer cells	Up	[12]
	TRF-25-R9ODMJ6B26	tRF-3	-	Upregulated in osteoporosis subjects	Up	[54]
	TRF-18-BS68BFD2	tRF-3	-	Upregulated in osteoporosis subjects	Up	[54]
	TRF-38-QB1MK8YUBS68BFD2	tRF-3	-	Upregulated in osteoporosis subjects	Up	[54]
Stem cell exhaustion	tRF/miR-1280	-	*JAG2*	Inhibits cell proliferation and tumour growth	Down	[55]
	tDR-000620	tRNA-1	-	Downregulated in cancer tissue	Down	[13]
Loss of proteostasis	5′-tiRNAVal	tiR-5	-	Inhibits the *FZD3*/Wnt/β-Catenin signalling pathway	Down	[41]
	tiRNAAla	tiR-5	eIF2α	Inhibits protein synthesis and triggers the phospho-eIF2a-independent assembly of stress granules (SGs)	Down	[44]
	tiRNACys	tiR-5	eIF2α	Inhibits protein synthesis and triggers the phospho-eIF2a-independent assembly of SGs	Down	[44]
	tRFVal	tRF-5	-	Induces the assembly of cytoprotective SGs	Down	[56]
Genomic instability	CU1276/tRF-3018	tRF-3	*RPA1*	Associated with Argonaute proteins, represses endogenous *RPA1*, suppresses proliferation and modulates the molecular response to DNA damage	Down	[34]
Deregulated nutrient sensing	rno-tRFi-Ser-25a	tiR-5	*Foxo1*	May play therapeutic roles through the FoxO signalling pathway	Down	[57]
	rno-tRF5-Ala-16a	tRF-5	-	May play therapeutic roles through the FoxO signalling pathway	Down	[57]
	rno-tRF5-Glu-29a	tRF-5	-	May play therapeutic roles through the FoxO signalling pathway	Down	[57]

### 3.1. Humans

Thus far, research on tRF expression in the human body has primarily investigated in cancer tissues. For example, in one study, qRT-PCR screened a total of 48 DE tRFs/tiRs in pancreatic cancer samples compared with adjacent normal tissue samples, including 46 upregulated tRFs/tiRs and 2 downregulated tRFs/tiRs [58]. A study investigating ovarian endometriosis [59] identified 19 upregulated and 5 downregulated tRFs, of which tiR-5s were the most common. In a study investigating high-grade serous ovarian cancer (HGSOC) [60], 2165 tRFs were found to be expressed in the sera of HGSOC patients and healthy controls. Of these tRFs, the 27 DE tRFs included 22 upregulated and 5 downregulated tRFs. The finding that tRFs are DE between healthy controls and cancer patients and the differences in expression between cancer tissue and adjacent normal tissue suggest that tRFs can be used as biomarkers for cancer diagnosis, prognosis and assessment of treatment efficacy. Although there is no direct evidence that tRFs directly affect aging or play a role in the aging pathway, these results suggest that model organisms could be used to study the functions and mechanisms of homologous tRFs in humans.

### 3.2. Mice

Mice are classic and practical research models of human aging and diseases [61, 62]. Recent research has found that tRFs are widely expressed in mouse tissues and organs. The different expression patterns of tRFs between the senescence-accelerated mouse prone 8 (SAMP8) model (a mouse model of premature senility used to study neurodegenerative diseases, such as Alzheimer's disease and Parkinson's disease) and the senescence-accelerated mouse resistant 1 (SAMR1) model (a mouse model that does not exhibit premature senility and is used as a control model) were examined by Zhang *et al*. [63]. Of the 570 tRF transcripts identified, 13 DE tRFs were detected [63]. Eight of the 13 DE tRFs, including AS-tDR-011775, AS-tDR-006835, AS-tDR-012690, AS-tDR-013428, AS-tDR-005058, AS-tDR-011389, AS-tDR-010789 and AS-tDR-011670, were confirmed by qRT-PCR [63]. The 8 confirmed DE tRFs had 110 potential target genes involved in the regulation of various functional brain signalling pathways, such as the synapse formation and synaptic vesicle cycle pathways, as determined by GO and KEGG enrichment analyses. These results suggest that the DE tRFs between SAMP8 and SAMR1 model mice are potential biomarkers and therapeutic targets for age-related brain diseases, such as Alzheimer's disease and Parkinson's disease [63]. In addition, miRNA data from the brains of young (6-month-old), middle-aged (14-month-old) and old (22-month-old) rats were used to analyse the expression levels of tRFs derived from 3'tRNAs and 5'tRNAs in the brains of rats of different ages [64, 65]. The results showed that the expression levels of 19 3'tRFs increased with age, while those of 24 5'tRFs did not [64]. The serum levels of specific subtypes of 5′tiRs markedly change with age[66]. These findings suggest that different types of tRFs can participate in the regulation of aging and age-related diseases through different expression patterns.

### 3.3. Drosophila melanogaster

For decades, *D. melanogaster* has been used as a classic model organism to explore the regulatory mechanisms of human aging and age-related diseases, such as cancer [67]. Studies investigating the relationships between tRFs and AGO proteins in *Drosophila* have shown that tRFs can directly bind and load onto AGO proteins and may target the 3' UTRs of mRNAs. Such studies have focused mainly on tRFs containing CCA sequences at their 3' ends and have revealed that these tRFs accumulate with age on Ago1 and Ago2 [14]. Small RNA libraries of *Drosophila* have revealed that Ago1 levels increase twofold (from 6% to 12%) from 3 days to 30 days and that Ago2 levels increase to an even greater extent (from 6% to 16%), indicating that tRFs change with age [14]. Our laboratory sequenced the small RNAs of *D. melanogaster* to explore the expression patterns of tRFs at different ages. The results showed that 487 tRFs/tiRs were DE between 7-day-old and 42-day-old *D. melanogaster*, including 271 upregulated and 127 downregulated tRFs/tiRs; 89 sequences showed no significant changes. Six tRFs that were upregulated with age were selected and verified by qRT-PCR. The results for tRF-Trp-CCA-014 and tRF-Val-CAC-020 were consistent with the sequencing results, while the results for the other four molecules (tRF-Ala-AGC-007, tRF-Ala-AGC-008, tRF-Val-TAC-003 and tRF-Val-TAC-004) were not. The two tRFs with consistent qRT-PCR and sequencing data (unpublished data) will be further studied in the future. As shown by these findings, tRFs in *D. melanogaster* may play important roles in the aging process, but more research is still needed to explore the functions and specific mechanisms of these molecules.

### 3.4. Caenorhabditis elegans

*C. elegans* is a classic model organism that is important for aging research due to its short lifespan and plentiful genetic resources. Studies investigating gene expression changes during aging in *C. elegans* have played important roles in elucidating the mechanisms of age-related phenomena and accelerating aging research progress [68]. Recently, total miRNA expression levels were found to decrease with aging in *C. elegans*, while tRF, ribosomal RNA (rRNA), small nucleolar RNA (snoRNA), and small nuclear RNA (snRNA) were found to accumulate during aging [69]. This interesting tRF expression pattern illustrates that tRFs may be correlated with aging and perform an important regulatory function in genetic senescence [69]. Although their biological functions remain largely unclear, tRFs may function as siRNAs or miRNAs to regulate biological processes.

**Figure 4.**
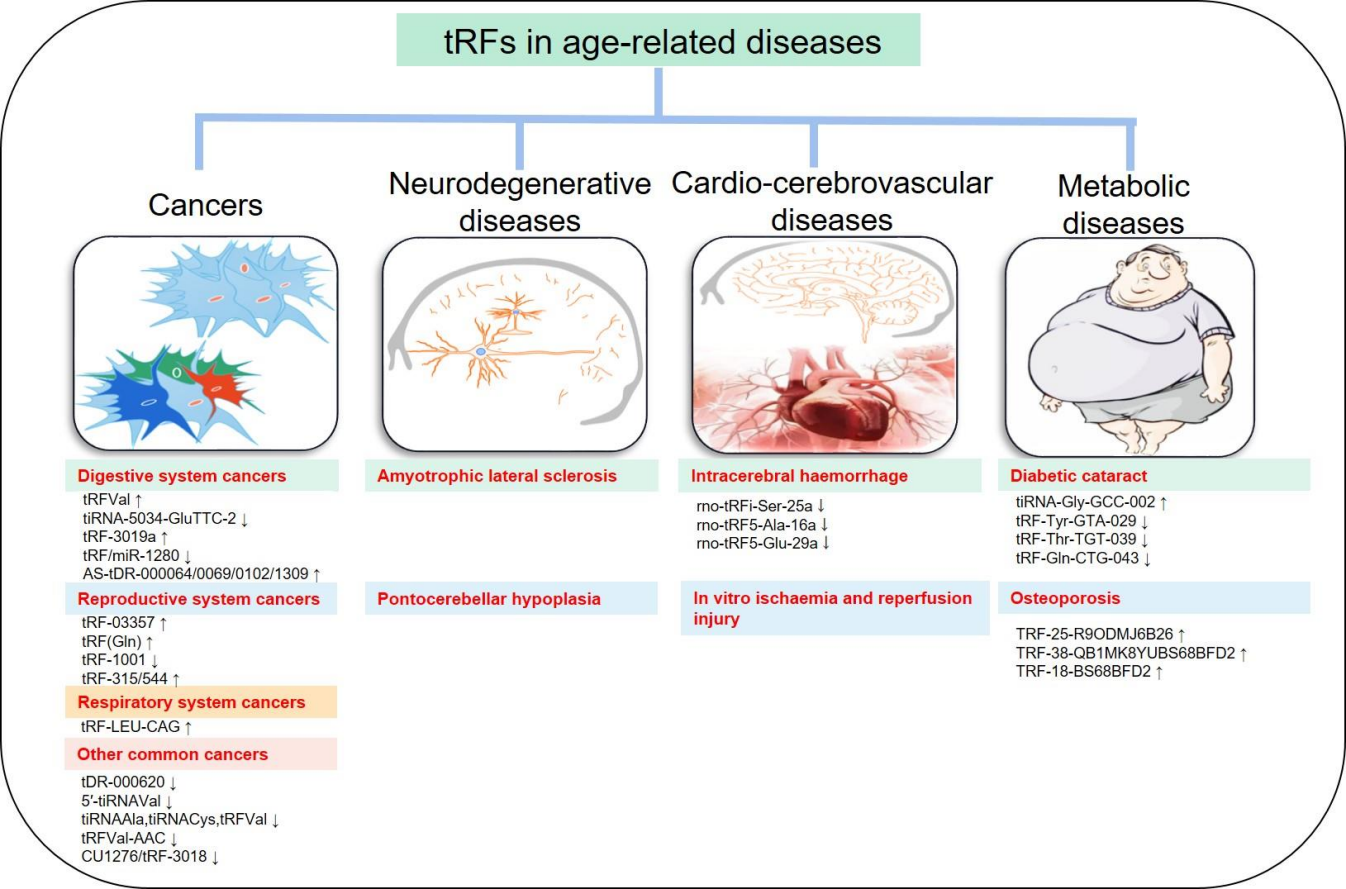
tRFs in age-related diseases. ?, upregulated in disease; ?, downregulated in disease.

Thus far, few studies have investigated tRFs in model organisms, but the expression patterns of tRFs have been found to differ among the existing model organisms. The expression levels of tRFs in mice, flies, and worms are closely related to aging, confirming that tRFs play important roles in brain functional signalling pathways in mice. The expression patterns and mechanisms of tRFs in other model organisms need to be studied in the future.

## 4. tRFs in age-related diseases

Aging is the greatest risk factor for age-related diseases, such as cancers (e.g., breast, colorectal, ovarian and pancreatic cancer), neurodegenerative diseases (Alzheimer's disease, Parkinson's disease, etc.), metabolic diseases and cardio-cerebrovascular diseases [1, 70-72]. In recent years, tRFs have been found to be abnormally expressed in the contexts of aging and multiple aging-related diseases and to participate in the regulation of disease processes (Fig. 4).

### 4.1. tRFs in the regulation of cancer

In cancer, senescence is an effective barrier preventing tumorigenesis. Identifying the key characteristics of senescence is an important step in eliminating senescent cells in aging tissues [73]. Inducing senescence and eliminating tumour cells via tumour immune response activation or autonomous apoptosis induction can also be used to eliminate senescent cells [74]. Recently, numerous studies have found that tRFs perform biological functions in various cancers and have revealed some regulatory mechanisms [75-78]. For example, downregulation of homeobox-containing 1 (*HMBOX1*) can promote cancer cell proliferation and migration in HGSOC [60]. In colorectal cancer, tRF/miR-1280 can inhibit cell proliferation and tumour growth by targeting Jagged 2 (*JAG2*) and inhibiting the Notch signal transduction pathway [55]. Differential expression of tRFs has been detected between some gastric cancer tissues and their adjacent normal tissues, suggesting that tRFs can be used as new biomarkers for cancer diagnosis [13, 58]. Therefore, tRFs are potential biomarkers for tumour prognosis prediction and determination of clinical treatment efficacy.

#### 4.1.1 tRFs in digestive system cancers

Digestive system cancers include mainly liver cancer, gastric cancer, pancreatic cancer and colorectal cancer [79]. The mortality rates of digestive system cancers are high; thus, research investigating the pathologic mechanisms of digestive system cancers is urgently needed. To date, several tRFs have been found to be involved in the regulation of digestive system cancers (Table 2).

Cancers are often associated with cell senescence, as senescence potently prevents tumorigenesis [73]. In digestive system cancers, tRFs regulate tumour size and inhibit tumour cell proliferation. Two such tRFs are tiRNA-5034-GluTTC-2 and tRF-3019a, which have opposite expression patterns between gastric cancer and normal tissues. Compared with normal tissues and cell lines, gastric cancer tissues and cell lines (HGC-27, AGS, BGC-823, SGC7901, and MGC-803) exhibit significantly downregulated expression of tiRNA-5034-GluTTC-2, and the expression level of this molecule is negatively correlated with tumour size, identifying tiRNA-5034-GluTTC-2 as a candidate molecular marker of gastric cancer [54]. In addition, the expression of TRF-3019a is upregulated in gastric cancer tissue and human gastric cancer cell lines (MGC-803, HGC-27, MKN-45, and AGS). Overexpression of TRF-3019a can promote proliferation, migration and invasion in gastric cancer cells, while knockdown of TRF-3019a expression can inhibit the proliferation of gastric cancer cells. Studies have shown that TRF-3019a regulates gastric cancer cells by directly regulating the tumour suppressor gene *FBXO47* [40]. Furthermore, the Notch pathway has often been found to be related to cell proliferation, differentiation, apoptosis, and other processes and is closely related to senescence. Notably, tRF/miR-1280 can reduce the proliferation and colony formation of patient-derived colorectal cancer cells by directly targeting the ligand *JAG2* in the Notch pathway [55], indicating that the Notch signalling pathway is involved in the regulation of colorectal cancer cells [55]. tRF/miR-1280 inhibits the growth and metastasis of colorectal cancer by inhibiting Notch signalling pathways that support the cancer stem-like cell (CSC) phenotype [55]. However, the mechanisms by which tRFs influence digestive system cancers are unclear and need to be further explored. High-throughput sequencing and qRT-PCR were used in one study to detect the expression of tRFs in pancreatic cancer tissues and adjacent normal tissues. In total, 48 tRFs and tiRs were identified in pancreatic cancer samples [59]. Some tRFs and tiRs were selected and verified by qRT-PCR, and the results were consistent with the sequencing results [59].

#### 4.1.2 tRFs in reproductive system cancers

Reproductive system cancers include mainly ovarian cancer, uterine cancer, prostate cancer and bladder cancer. In recent years, it has also been reported that tRFs are involved in the regulation of reproductive system cancers (Table 2). tRFs are abnormally expressed in ovarian cancer tissues [83]; for example, the expression levels of TRF-03357 and TRF-03358 in HGSOC patients are significantly higher than those in healthy subjects [60]. TRF-03357 downregulates the expression of the *HMBOX1* gene and promotes the proliferation, migration and invasion of HGSOC cells [60]. In a study investigating ovarian endometriosis, small RNA sequencing (smRNA-SEQ) of endometrial tissue from 3 pairs of ovarian endometriosis samples and normal endometrium samples was performed. In total, 19 upregulated and 5 downregulated tRFs were identified in the endometrium. The most common KEGG and GO functional clusters of tiR-5 suggested that tRFs may play a role in the pathogenesis of endometriosis [84]. Furthermore, abnormal expression of tRFs has been observed in not only the female reproductive system but also the male reproductive system; tRFs are highly expressed in testicular germ cell tumours, and the highly expressed tRFs are derived mostly from the 5' ends of mature tRNAs, primarily tRNA-Glu-GAG and tRNA-Asp-GAY [5]. Similarly, in prostate cancer, tRFs can accelerate the cell cycle. For example, tRF-1001 is a 3' terminus-derived tRF produced by the cleavage of Ser-TGA under the action of ribonuclease Z, and its expression is positively correlated with the proliferation of prostate cancer cells [5]. Overexpression of TRF-1001 can promote the G2-to-M phase transition of prostate cancer cells [5]. Systematic research investigating tRFs in the reproductive system will provide new diagnostic and treatment ideas for reproductive system cancers.

**Table 2:** tRFs in cancers.

	Cancer type	tRF name	Type	Targets	Function	Dys-regulation	Ref.
Digestive system cancers	Liver cancer	tRF^Val^	tRF-5	-	Upregulated in cancer tissue	Up	[80]
	Gastric cancer	tiRNA-5034-GluTTC-2	tiR-5	-	Downregulated in cancer tissue, and the expression level is inversely proportional to tumour size	Down	[54]
		tRF-3019a	tRF-3	*FBXO47*	Upregulated in cancer tissue and regulates the tumour suppressor gene *FBXO47*	Up	[40]
	Colorectal cancer	tRF/miR-1280	-	*JAG2*	Inhibits cell proliferation and tumour growth	Down	[55]
	Pancreatic cancer	AS-tDR-000064	Leu-AAG-1-1	-	Upregulated in cancer tissue	Up	[58]
		AS-tDR-000069	Gln-CTG-1-1	-	Upregulated in cancer tissue	Up	[58]
		AS-tDR-000102	Ala-CGC-1-1	-	Upregulated in cancer tissue	Up	[58]
		AS-tDR-001391	Pro-CGG-1-1	-	Upregulated in cancer tissue	Up	[58]
Reproductive system cancers	HGSOC	tRF-03357	-	*HMBOX1*	Might partially promote the proliferation, migration and invasion of ovarian cancer	Up	[60]
	Cervical cancer	tRF(Gln)	tRF-5	-	Inhibits protein translation without the need for complementary target sites on mRNA	Up	[47]
	Prostate cancer	tRF-1001	tRF-1	-	Regulates cell proliferation	Down	[5]
		tRF-315	tRF-5	-	Predicts poor progression-free survival (PFS)	Up	[81]
		tRF-544	tRF-3	-	Upregulated in cancer tissue	Up	[81]
Respiratory system cancers	NSCLC	tRF-LEU-CAG	tiR-5	AURKA	Related to cancer cell proliferation	Up	[12]
Other common cancers	Breast cancer	tDR-000620	tRNA-1	-	Downregulated in cancer tissue	Down	[13]
		5′-tiRNAVal	tiR-5	-	Inhibits the *FZD3*/Wnt/β-Catenin signalling pathway	Down	[41]
	Osteosarcoma	tiRNAAla	tiR-5	eIF2α	Inhibits protein synthesis and triggers the phospho-eIF2a-independent assembly of stress granules (SGs)	Down	[44]
		tiRNACys	tiR-5	eIF2α	Inhibits protein synthesis and triggers the phospho-eIF2a-independent assembly of SGs	Down	[44]
		tRFVal	tRF-5	-	Induces the assembly of cytoprotective SGs	Down	[56]
	Clear-cell renal cell carcinoma (ccRCC)	tRF4-Val-AAC	tiR-5	-	Downregulated in ccRCC tissues	Down	[82]
	B-cell lymphoma	CU1276/tRF-3018	tRF-3	*RPA1*	Associated with Argonaute proteins, represses endogenous *RPA1*, suppresses proliferation and modulates the molecular response to DNA damage	Down	[34]

#### 4.1.3 tRFs in respiratory system cancers

tRFs are also involved in the regulation of respiratory cancers (Table 2). tRF-LEU-CAG is upregulated in human NSCLC tissues, cell lines (the human cell lines SPCA-1, 95-D, H1650, A549, H129, PC-9 and H23) and patient serum compared to normal samples [12]. However, AURKA can inhibit the expression of tRF-LEU-CAG [12]. AURKA is abnormally expressed in various tumours and participates in a variety of important cellular signal transduction pathways; these pathways can directly or indirectly activate kinase oncoproteins and tumour suppressor proteins to promote tumour development and can regulate mitosis by binding the centrosome [85, 86]. The proliferation ability of human H1299 cells is decreased upon tRF-LEU-CAG inhibitor treatment, indicating that tRF-LEU-CAG is related to NSCLC cell proliferation [12]. Therefore, it is speculated that tRF-LEU-CAG may also be involved in the regulation of the course of NSCLC. In addition, studies investigating miR-181A-5p, miR-146-5p, miR-137 and miR-32 in NSCLC have shown that miRNA expression can be associated with the occurrence of NSCLC [86-88]. Thus, miRNAs and tRFs may co-regulate gene expression.

#### 4.1.4 tRFs in other common cancers

tRFs are also involved in the regulation of cancers other than the above-mentioned cancers (Table 2). Similar to patterns described thus far, these tRFs show differential expression in cancer tissues compared to normal tissues, exhibiting downregulation in breast cancer, osteosarcoma, clear-cell renal cell carcinoma, and B-cell lymphoma. The regulatory mechanisms of several tRFs, including 5°-tiRNAVal [41], tiRNAAla [44], tiRNACys [44], tRFVal [56], and CU1276/tRF-3018 [34], have been unravelled. YBX-1 is an RNA-binding protein that plays very important roles in tumorigenesis, development, metastasis, cancer treatment and drug resistance prediction and is abnormally expressed in various cancers [36]. Recent studies have reported that endogenous tRFs can compete with carcinogenic transcription factors, such as AKT, EIF4G1 and HMGA1, to bind YBX-1. Knockout of YBX-1 can promote the growth of tumour cells [36]. In addition, tRF-CU1276 inhibits the expression of endogenous *RPA1* (which is involved in DNA replication and repair) [89] to reduce the efficiency of DNA replication in B-cell lymphoma cells, thus inhibiting tumour cell proliferation [34]. In summary, tRFs play important roles in the courses of various cancers, and the existing evidence provides insights for further studies on diagnosis and treatment.

### 4.2. tRFs in neurodegenerative diseases

Neuropathic diseases, including Alzheimer's disease and Parkinson's disease, are affected by genetic and environmental factors [90]. The incidence of these brain-related diseases tends to increase with age, and the diseases are accompanied by dynamic changes in regulatory factors. Similarly, recent studies have found that tRFs dynamically change with aging in mammalian brain cells [64, 91]. tRFs in the prefrontal cortex, cerebrospinal fluid and serum can be used as non-invasive biomarkers of Parkinson's disease [92]. tRFs are known to participate in the regulation of neurodegenerative diseases. For example, TRHs (similar to tiRs in classification) have been identified in the context of human amyotrophic lateral sclerosis (ALS) and can provide protection to motor neurons in ALS [93]. Loss of protective TRHs or the interaction between protective TRHs and pathogenic RNA repeats may be involved in the regulation of ALS progression [93]. Defects in tRNA processing lead to tRF accumulation, which may trigger the occurrence of neurodegenerative diseases [94, 95]. The formation of tRFs is closely related to ANG. Mutant ANG causes abnormal shearing of tRNA and abnormal aggregation of tRFs [94, 95]. A mutation in the *CLP1* gene, which encodes the RNA kinases spliced by tRNA, has been found in patients with pontocerebellar hypoplasia (PCH). PCH is a heterogeneous hereditary neurodegenerative disease characterized by developmental damage in different parts of the brain. Epigenetic abnormalities and neuromuscular defects are observed in PCH patients and animal models (mice and zebrafish) with *CLP1* deficiency [96-98]. *CLP1* mutation leads to depletion of mature tRNA and accumulation of unspliced precursor tRNA in patient-derived neurons [96-98]. Although the exact mechanism of the interaction among *CLP1* activity, tRNA splicing and tRF function is unclear, these findings provide a basis for further studies concerning tRNA metabolic abnormalities and neurodegeneration.

Currently, the mechanisms by which tRFs exert their confirmed regulatory effects in neurodegenerative diseases are unclear. Further research investigating tRFs could elucidate these mechanisms and enable tRFs to be applied for the diagnosis and treatment of neurodegenerative diseases.

### 4.3. tRFs in cardio-cerebrovascular diseases

Cardio-cerebrovascular diseases include cardio-vascular and cerebrovascular diseases. These diseases are severe threats to humans, especially those aged over 50 years, and have high prevalence, high morbidity and high mortality [99]. Common cardio-cerebrovascular diseases include coronary heart disease and stroke. Recent developments in tRF research have revealed that tRFs also play roles in cardio-cerebrovascular diseases.

#### 4.3.1 Cerebral ischaemia and reperfusion injury

tRF dysregulation has been demonstrated to be related to ischaemia. tRNAVal- and tRNAGly-derived small RNA fragments were found to be the most abundant fragments in a rat model of cerebral ischaemia [100]. Importantly, upregulation of tRNAVal- and tRNAGly-derived fragments inhibited cell proliferation, migration and tube formation. These results indicate that tRFs play a role in cerebral ischaemia [100]. Furthermore, oxygen-glucose deprivation (OGD) was found to induce tRNA cleavage and tiR production in PC12 rat neural cell lines [35]. OGD significantly upregulated the production of tiRs a few minutes after reperfusion. The degree of tiR generation dramatically increased after 1 and 3 h of reperfusion and decreased after 6 h. In the context of reperfusion injury, rapidly measurable biomarkers are needed to aid in immediate treatment decisions for patients with acute stroke symptoms. tiRs are potential biomarkers of cell damage and indexes of therapeutic efficacy that can be used in the context of this type of injury.

#### 4.3.2 Intracerebral haemorrhage

tRFs are also involved in the medical treatment of cardiovascular diseases. Buyang-Huanwu decoction (BYHWD) is a centuries-old Chinese medicine that promotes the recovery of neurological function after intracerebral haemorrhage (ICH), but its therapeutic mechanism is unclear [57]. BYHWD treatment obviously improves behavioural endpoints during the recovery phase after ICH, and 3 tRNAs (rno-tRFi-Ser-25a, rno-tRF5-Ala-16a and rno-tRF5-Glu-29a) have been found to be markedly regulated by BYHWD treatment and validated by qRT-PCR [57].

Although tRFs are known to participate in cardiovascular disease regulation, the related mechanisms require more research. Nevertheless, the existing evidence can provide ideas for the treatment of this type of disease.

### 4.4. tRFs in metabolic diseases

Metabolic diseases are common complications after liver transplantation and are risk factors for cardiovascular disease and death [101]. Common metabolic diseases include diabetes, hypoglycaemia, gout and osteoporosis.

#### 4.4.1 Diabetic cataract

Diabetes is among the most common diseases in elderly individual, and the streptozotocin (STZ)-induced diabetic cataract (DC) mouse model is often used in human diabetes research [102]. In the past few years, RNA sequencing of rat lens epithelial cells from DC rats identified 213 DE tRFs, including 111 upregulated and 102 downregulated tRFs; five of these tRFs/tiRs were verified by qRT-PCR. This research produced the first expression profile of tRFs/tiRs in DC rats and found that four types of tRFs/tiRs (tiRNA-Gly-GCC-002, tRF-Tyr-GTA-029, tRF-Thr-TGT-039 and tRF-Gln-CTG-043) are involved in the pathogenesis of DC. The identification of these tRFs/tiRs may provide a new perspective for further studies investigating the exact mechanism of DC [103].

#### 4.4.2 Osteoporosis

Osteoporosis is a common metabolic disease in the elderly population that becomes increasingly common with age. Studies have found that tRFs are also associated with this disease. A study detecting small RNAs in the plasma of normal and osteoporotic patients showed that TRF-25 (TRF-25-R9ODMJ6B26), TRF-38 (TRF-38-QB1MK8YUBS68BFD2) and TRF-18 (TRF-18-BS68BFD2) were highly expressed in osteoporotic patient plasma and may be biomarkers of osteoporosis [104].

Diabetes and osteoporosis are both common diseases in older age groups, and research concerning tRFs has helped us better understand these two types of diseases. Further in-depth studies of tRFs will likely reveal additional metabolic diseases associated with tRFs and provide new tools and ideas for the diagnosis and treatment of such diseases. Similarly, further research will likely clarify the roles of tRFs in aging-related diseases and enable the development of additional applications of tRFs for these conditions.

### 4.5. tRFs as biomarkers of age-related diseases

tRFs associated with aging-related diseases might serve as targets for gene therapy or as disease biomarkers. Studies investigating tRFs must assess whether tRFs can be used as predictive biomarkers of diseases by detecting their sensitivity and specificity.

#### 4.5.1 i-tRF-GlyGCC in chronic lymphocytic leukemia (CLL)

CLL affects mostly adults, usually elderly people, and the clinical features of CLL vary considerably [105]. A study investigating the tRF i-tRF-GlyGCC in CLL showed that although the expression of i-tRF-GlyGCC cannot be used to distinguish non-CLL patients from blood donors, it is associated with CLL prognosis [106]. During the study, 29 of 81 people died due to CLL, and the estimated median overall survival (OS) time was 73 months. A Kaplan-Meier analysis revealed that i-tRF-GlyGCC-positive CLL patients had shorter OS times than i-tRF-GlyGCC-negative CLL patients. Thus, elevated levels of i-tRF-GlyGCC were related to poor OS in patients with CLL. Univariate bootstrap Cox regression analysis confirmed these results by demonstrating a higher hazard ratio (HR) of 3.40 among patients overexpressing i-tRF-GlyGCC than among those not overexpressing i-tRF-GlyGCC [105]. As uncovered by this analysis, Binet A-staged patients overexpressing i-tRF-GlyGCC appeared to have worse OS than those with lower expression levels of this molecule; similar results have been found in patients with CLL at Binet stages B and C and Rai stages II and IV [106]. Thus, i-tRF-GlyGCC is a potential prognostic biomarker of CLL.

#### 4.5.2 tiRNA-5034-GluTTC-2 in gastric cancer

One study found that the expression of tiRNA-5034-GluTTC-2 was downregulated in gastric cancer tissues, plasma and gastric cancer cell lines and that lower levels of tiRNA-5034-GluTTC-2 were related to larger tumour size; conversely, greater expression was associated with a smaller tumour diameter [54]. The expression levels of tiRNA-5034-GluTTC-2 were significantly downregulated in 73.3% (63/86) of gastric cancer tissues compared with non-tumorous tissues. Thus, tiRNA-5034-GluTTC-2 was found to be a new gastric cancer diagnostic biomarker that can be used to distinguish gastric cancer tissues from normal tissues. The sensitivity and specificity were 73.3% and 46.5%, respectively, and the false-negative and false-positive rates were 26.7% and 53.5%, respectively. The negative predictive value (NPV) and positive predictive value (PPV) were 73.4% and 65.2%, respectively. With the combined use of tissue and plasma tiRNA-5034-GluTTC-2 as a biomarker, the sensitivity and specificity were 84.7% and 92.8%, respectively. In addition, the OS in the lower-tiRNA-5034-GluTTC-2-expression group was significantly lower than that in the higher-expression group [54]. Therefore, tiRNA-5034-GluTTC-2 has great potential as a biomarker of gastric cancer.

#### 4.5.3 5'-tiRNAVal in breast cancer

The area under the curve (AUC) of the receiver operating characteristic (ROC) curve is a standard evaluation parameter, and a greater AUC value is better than a smaller AUC value. One study evaluated the discriminative power of 5'-tiRNAVal between control tissues and breast cancer tissues by determining the ROC curve AUC [41]. The results showed that the ROC curve AUC of 5'-tiRNAVal in the differentiation of all breast cancer patients from healthy controls was 0.756, with a cut-off value of 5.433, a sensitivity of 90.0%, and a specificity of 62.7%. Different sensitivity and specificity values were obtained for differentiation of breast cancer tissues of various tumour-node-metastasis (TNM) stages from healthy control tissues; a cut-off value of 5.433 yielded a sensitivity of 85.0% and a specificity of 51.9% during the early stages [41]. These results suggest that 5'-tiRNAVal is a diagnostic biomarker of breast cancer.

Biomarkers can be used for disease diagnosis and staging and for evaluation of the safety and efficacy of a new drug or therapy in a target population. Research investigating the roles of i-tRF-GlyGCC, tiRNA5034-GLUTC-2 and 5'-tiRNAVal in cancers has provided new biomarkers for the diagnosis of the three corresponding cancers. In addition, a few studies have suggested that other tRFs are potential markers of diseases, but more in-depth illustrative studies are needed [60, 92]. Biomarkers can aid in disease diagnosis, but many more datasets are required to determine how well tRFs perform as prognostic biomarkers and whether they can serve as therapeutic targets.

## 5. Methods and tools in tRF research

### 5.1. Identification of tRFs

Research concerning tRFs has focused mainly on identification, functional verification and exploration of the regulatory mechanisms. Thus far, various techniques have been widely used to identify tRFs in primary tumour tissues and cells. High-throughput sequencing [5] and next-generation sequencing [12] can be used to screen DE tRFs in different tissues. Northern blot [12, 54] and qRT-PCR [5, 12, 54] analyses can be used to verify the authenticity of tRF expression levels in sequencing results. In most studies, the total RNA extracted from samples for detection of tRFs must be pretreated to remove modifications that interfere with PCR, such as RNA methylation [54, 107]. Stem-loop primers with high specificity are used to reverse-transcribe each tRF in the pretreated RNA into cDNA for subsequent experiments or preservation [108].

### 5.2. Bioinformatics analysis of tRFs

Following the development of high-throughput RNA sequencing technology and the application of various modern biological tools, tRF databases have been established for many species and can provide references for further research (Table 2). tRFdb [23] was the first database established and is the most comprehensive and commonly used database in studies investigating tRFs. This database includes 16738 tRFs from more than 200 libraries obtained from 8 species *(Rhodobacter sphaeroides, Schizosaccharomyces pombe, Drosophila, C. elegans, Xenopus*, zebrafish, mice and humans). Other databases containing basic information regarding tRFs include MINTbase [109], YM500v3 [110] and tRFinCancer [111]. MINTbase [109] contains tRF molecular sequence and expression information as well as the original tRNA and corresponding genome information. Thus far, most studies investigating tRFs have been related to the regulatory mechanisms of tRFs in cancer. Cancer-related tRF data are collected in YM500v3 [110] and tRFinCancer [111]. YM500v3 contains not only tRF datasets but also more than 8000 datasets from smRNA-SEQ and comprehensive analyses of the results of miRNA studies investigating various cancers. tRFexplorer provides researchers with the expression profiles of tRNA-derived ncRNAs from nine different cancer types (including leukaemia, colon cancer, lung cancer, central nervous system cancers, kidney cancer, melanoma, ovarian cancer, breast cancer and prostate cancer) derived from The Cancer Genome Atlas (TCGA) and National Cancer Institute-60 (NCI-60) data [112]. In addition, TargetScan (www.targetscan.org/) and miRanda (www.microrna.org/microrna/home.do) can be used to predict the target genes of tRFs and tiRs [58].

Online tRF network tools, such as tRF-Browser [111], High-throughput Annotation of Modified Ribonucleotides (HAMR) [113], tRF2Cancer [111], tRFinCancer [111], tDRmapper [114], sRNAtools [115] and MINTmap [116, 117], can be used for tRF identification and functional annotation (Table 2). Most RNA molecules contain many nucleotide modifications, which can hinder the identification and recognition of tRFs. tRF-Browser and HAMR can be used to determine the original sites of tRFs, elucidate the distributions of chemically modified sites in tRFs and identify tRFs in numerous smRNA-SEQ data pools. tRF2Cancer [111] and tRFinCancer [111] are two tools from the same tRFinCancer database that contain small RNA deep sequencing data; these data can be used to obtain the tRF expression levels in various cancers. High-throughput sequencing has helped identify some tRFs, and sRNAtools [115] can be used to identify and annotate sncRNA functions from high-throughput sequencing data.

In summary, these databases and tools help researchers identify and understand the roles of new tRFs in cancers and other human diseases. These databases also promote understanding of the occurrence, development, diagnosis and prognosis of diseases.

### 5.3. Investigation of the functions and regulatory mechanisms of tRFs

The existing research on the functions and mechanisms of tRFs can be divided into *in vivo* and *in vitro* experiments, and both gain-of-function and loss-of-function experiments have been conducted [77]. *In vivo*, the expression of tRFs has typically been compared between different tissues or different regions. For example, in one study, 48 DE tRFs and tiRs were detected between pancreatic cancer tissues and adjacent normal tissues, including 46 upregulated tRFs (or tiRs) and 2 downregulated tRFs (or tiRs). To investigate the effect of tRF/miR-1280 on colorectal cancer, HCT116 and HCT116-tRF/miR-1280 colorectal cancer cells were transplanted into athymic nude mice to establish xenograft tumours, and the tumour volume and tumour cell metastasis were observed [37]. Thus far, no research has investigated the functions and mechanisms of tRFs via knockdown or overexpression of tRFs in model organisms, such as flies. *In vitro* research has primarily been conducted in cells; when target tRFs are screened by sequencing, corresponding cell lines can be used to verify the accuracy of the sequencing results *in vitro* [55, 60]. One study knocked down the expression of tRFs that form the factors ANG and Dicer and detected known tRFs to identify whether tRF formation is related to ANG/Dicer formation [37]. For some tRFs, loss- or gain-of-function experiments can be performed; siRNAs can be designed to knock down the expression of tRFs, or cells can be transfected with a synthetic sequence to verify the effects of overexpression in the cells [17, 60, 106].

Northern blot and qRT-PCR analyses are traditional methods used for nucleic acid identification and can also be used for tRF identification. After identification, collection and analysis of tRF data are also very important parts of tRF research. The existing databases are not comprehensive, and the analyses have not been systematic; therefore, a unified database is needed to organize tRF data. Following the discovery of new tRF mechanisms, the development of new technology could help reveal the functions and regulatory mechanisms of tRFs.[Table T3-ad-12-5-1304]

**Table 3 T3-ad-12-5-1304:** Databases and tools used in tRF research.

Tool	Description	URL	Ref.
tRFdb	Contains information regarding tRFs in 8 different organisms	Http://genome.bioch.virginia.edu/trfdb	[23]
MINTbase	Integrates four types of information regarding tRFs	Http://cm.jefferson.edu/MINTbase/	[109]
YM500v3	Contains smRNA-SEQ data from human cancer research	Http://ngs.ym.edu.tw/ym500/	[110]
tRFinCancer	Enables viewing of the expression of tRFs in multiple cancer types	Http://rna.sysu.edu.cn/tRFfinder/	[111]
tRF2Cancer	Useful for accurate identification of tRFs from small RNA deep sequencing data and determination of tRF expression levels in multiple cancers	Http://rna.sysu.edu.cn/tRFfinder/	[111]
tRFexplorer	Allows researchers to study the potential biological effects of tRFs without any direct experimental evidence	Https://trfexplorer.cloud/	[112]
tRF-Browser	Can be used to determine the sites of origin and distributions of chemical modification sites in tRFs, including m5C, 2, O-Me, Ψ and m6A sites	Http://rna.sysu.edu.cn/tRFfinder/	[111]
HAMR	Identifies potential signatures of nucleotide modifications	Http://wanglab.pcbi.upenn.edu/hamr	[113]
tDRmapper	Developed as an alignment tool for mapping, naming, quantifying and graphically visualizing novel tRFs	Https://github.com/sararselitsky/tDRmapp	[114]
sRNAtools	Can be used in conjunction with high-throughput sequencing to identify and functionally annotate sncRNAs, including tRFs	Https://bioinformatics.caf.ac.cn/sRNAtools	[115]
MINTmap	Can be used for mitochondrial and nuclear tRF mapping	Https://github.com/TJU-CMC-Org/MINTmap/	[116]

## 6. Summary and prospects

Aging involves the participation, cooperation, and influence of various factors and mechanisms. At the genetic level, research on the effects of tRFs in aging is still scarce. RNA degradation often produces random fragments, but tRFs are not random fragments generated by tRNA, and their cutting positions have obvious regularity. However, the molecular mechanism of tRF production is only partially understood. With the development of high-throughput sequencing technology, increasing numbers of tRFs have been found. In addition, in recent years, increasing numbers of reports have revealed that tRFs are related to many types of cancer, and cancers are typical aging-related diseases. Some mechanisms of tRFs and tiRs in cancer have been clarified, showing that studies on tRFs can expand the field of disease research and provide new perspectives in this field. The identification of tRFs as a new type of sncRNA not only broadens the research field of sncRNAs but also reveals that tRNAs participate in gene regulation as dynamic factors.

tRFs play important regulatory roles in gene transcription and translation, cell proliferation, cell stress responses and cancer. Research concerning their biological functions and mechanisms has begun, but their regulatory mechanisms still need to be fully characterized. Animal models can aid in the discovery of new ncRNAs and in elucidation of the phenotypic significance of ncRNAs. For example, *Drosophila* can be used to study tRFs associated with aging. Model organisms can be used to fully screen the DE tRFs during aging and are convenient tools for investigation of tRF-related disease mechanisms *in vivo*. Model organism research also supports the translation of experimental results to clinical medicine. In addition, in functional and mechanistic studies, model organisms can more directly reflect the effects of tRFs on organisms than *in vitro* systems. However, no studies have directly knocked down or overexpressed tRFs in model organisms, perhaps because tRFs are highly modified and easily affected by other factors in the body. tRFs, a new type of sncRNA, may have functional overlap with other molecules, and there may be some interactions and functional intersections of tRFs with miRNAs, lncRNAs and other ncRNAs; these possibilities need to be investigated in further experiments. Furthermore, there is currently no unified naming system for tRFs and tiRs, and few new technologies have been applied to research these molecules. Therefore, it is necessary to establish effective research methods to systematically study the structures and mechanisms of tRFs and tiRs. In the future, an increasing number of tRFs will be recognized and identified, and further research investigating tRFs will support additional applications for aging and aging-related diseases.
